# Direct mechanical thrombectomy vs. intravenous alteplase plus mechanical thrombectomy in acute ischemic stroke with anterior circulation tandem occlusions

**DOI:** 10.3389/fsurg.2025.1536912

**Published:** 2025-04-22

**Authors:** Zhonghui Yang, Guang Zhang, Qiaowei Wu, Yujing Zhu, Shancai Xu, Huaizhang Shi

**Affiliations:** Department of Neurosurgery, The First Affiliated Hospital of Harbin Medical University, Harbin, Heilongjiang, China

**Keywords:** direct mechanical thrombectomy, acute ischemic stroke, intravenous alteplase, anterior circulation, tandem occlusion

## Abstract

**Background and purpose:**

Tandem occlusion is a significant risk factor for poor outcomes following intravenous thrombolysis. The necessity of bridging therapy [intravenous thrombolysis prior to mechanical thrombectomy (MT)] for patients with tandem occlusion remains controversial. This study assessed the safety and efficacy of direct MT vs. bridging therapy in patients with tandem occlusions in the anterior circulation.

**Methods:**

This retrospective study enrolled patients with anterior circulation tandem occlusions treated with either direct mechanical thrombectomy (MT-alone group) or intravenous alteplase thrombolysis followed by MT (bridging group) between January 2019 and March 2022. The primary outcome was prespecified as a favorable outcome [modified Rankin Scale (mRS) score of 0–2] at 90 days. Secondary outcomes included successful reperfusion, overall mortality at 90 days, and rates of symptomatic intracranial hemorrhage (SICH) and asymptomatic intracranial hemorrhage (aSICH).

**Results:**

A total of 110 patients were enrolled, with 49 in the MT-alone group and 61 in the bridging group. A favorable outcome (mRS score of 0–2) at 90 days was achieved in 25 patients (51.0%) in the MT-alone group and in 34 patients (55.7%) in the bridging group, showing no significant difference between the groups, with an adjusted odds ratio (aOR) of 1.17 (95% CI, 0.47–2.90; *P* = 0.743). The incidence of aSICH was higher in the bridging group than in the MT-alone group [31.1% vs. 14.3%; aOR, 2.86 (95% CI, 1.04–7.88); *P* = 0.042]. Rates of successful reperfusion, overall mortality at 90 days, and SICH were similar between the groups. Multivariate analysis showed that a lower baseline National Institutes of Health Stroke Scale (NIHSS) score (*P* = 0.005), intraprocedural tirofiban administration (*P* = 0.012), and internal carotid artery stent implantation (*P* = 0.040) were associated with a favorable outcome at 90 days.

**Conclusion:**

This study found no evidence that prior intravenous thrombolysis affects clinical or imaging outcomes in patients with acute ischemic stroke due to anterior circulation tandem occlusions after endovascular thrombectomy. Bridging therapy may be associated with an increased rate of aSICH. Intraprocedural tirofiban administration, stent implantation, and a lower baseline NIHSS score were associated with favorable outcomes.

## 1 Introduction

Anterior circulation tandem occlusion was defined as an intracranial anterior circulation large vessel occlusion [distal intracranial internal carotid artery (ICA) or M1-M2 segment of the middle cerebral artery] along with ipsilateral high-grade cervical ICA stenosis or occlusion (complete occlusion or stenosis ≥90% as per the North American Symptomatic Carotid Endarterectomy Trial) (1–3). Anterior circulation tandem occlusion occurs in approximately 15%–20% of patients with acute ischemic stroke (4). Due to its complex pathophysiology, managing such lesions presents a significant clinical challenge. Tandem occlusion is a notable risk factor for poor outcomes following intravenous thrombolysis (1, 5), and published studies have demonstrated the safety and efficacy of endovascular mechanical thrombectomy (MT) for tandem occlusion (1–4). For patients with acute large artery anterior circulation stroke, multiple studies have reported the benefits of bridging therapy (intravenous thrombolysis prior to MT) (6–10). However, recent studies suggest that, for patients with acute large vessel occlusive stroke, direct mechanical thrombectomy may be non-inferior to bridging therapy in terms of recanalization and outcomes (10, 11). Previous randomized controlled studies, however, did not specifically describe or summarize outcomes for patients with tandem lesions, leaving a lack of sufficient evidence on whether anterior circulation tandem occlusions benefit from bridging therapy (11–13). This retrospective study aimed to compare clinical outcomes in patients with anterior circulation tandem occlusions treated with bridging therapy vs. those treated with MT alone.

## 2 Materials and methods

### 2.1 Study design and patients

This retrospective, observational cohort study assessed the safety and efficacy of direct MT vs. bridging therapy in patients with anterior circulation tandem occlusions from the First Affiliated Hospital of Harbin Medical University between January 2019 and March 2022. We screened 1,201 patients with acute anterior circulation stroke who had received endovascular treatment. Among them, 110 patients with tandem occlusions meeting the inclusion criteria were enrolled in our study. The patients included in the study received either MT alone (MT-alone group) or intravenous alteplase followed by MT (bridging group).

**Figure 1:**
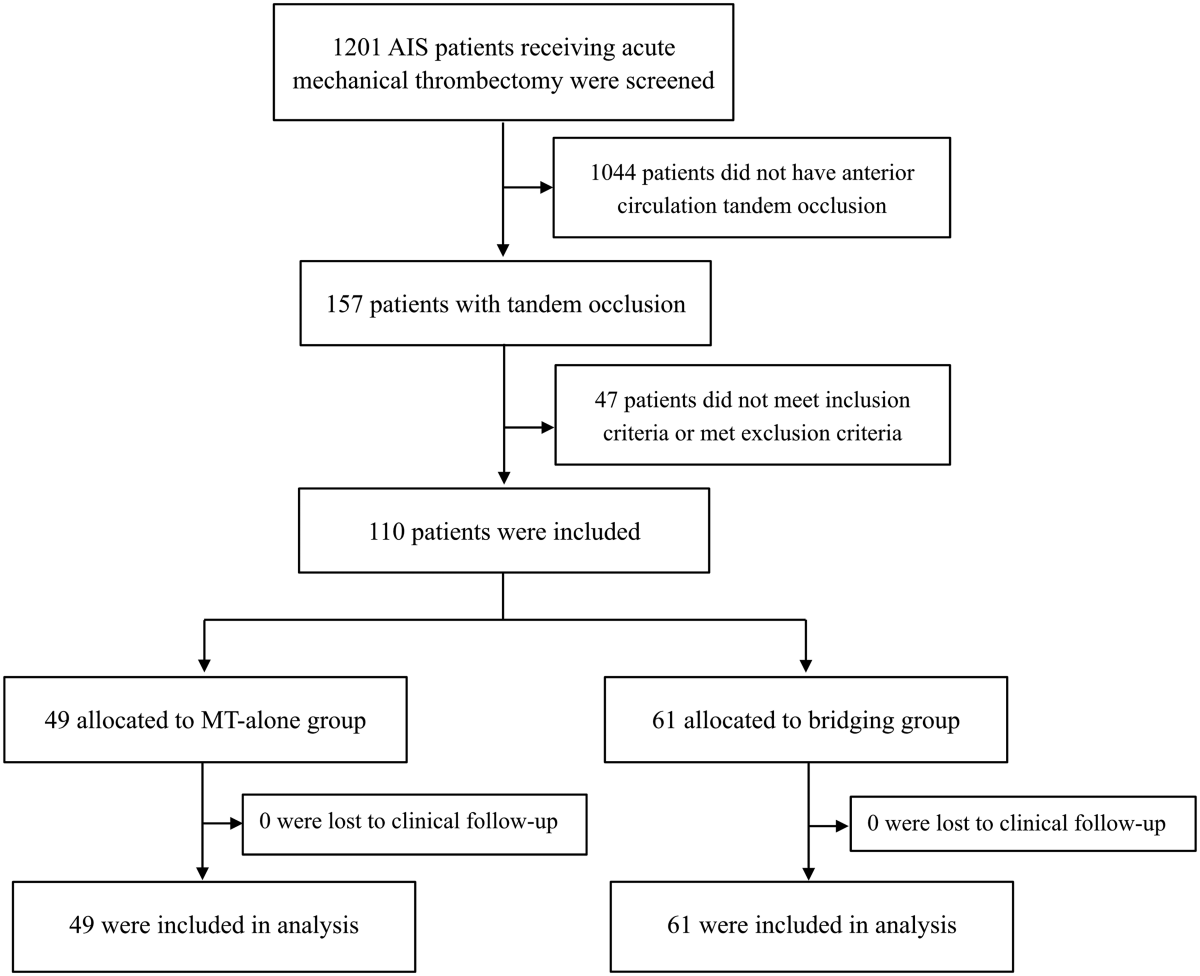
Study flowchart. AIS, acute ischemic stroke; MT, mechanical thrombectomy.

To be eligible for inclusion in this study, the patients had to meet the following inclusion criteria: (1) patients were 18 years of age or older; (2) had disabling symptoms, defined as a National Institutes of Health Stroke Scale (NIHSS) score ≥6 (13); (3) based on non-contrast computed tomography (CT), patients with an Alberta Stroke Program Early CT Score (ASPECTS) ≥6; (4) patients with anterior circulation tandem occlusions as shown on computed tomographic angiography (CTA) or digital subtraction angiography (DSA), that could be treated with intravenous alteplase within 4.5 h after symptom onset; (5) patients receiving intravenous thrombolysis met the indications for alteplase administration, with no contraindications; (6) all patients received endovascular treatment within 6 h of acute stroke onset. Patients were excluded from the study if they met any of the following exclusion criteria: (1) angiographic results after thrombolysis indicated vascular recanalization without the need for further endovascular intervention; (2) patients received intra-arterial alteplase or urokinase as rescue therapy after reperfusion failure; (3) patients with an mRS score >2 before symptom onset; (4) patients who received treatment with other intravenous thrombolytic drugs, such as urokinase, were excluded to reduce the potential impact on the outcomes. The study flowchart is shown in Figure 1. Our study was approved by the Ethics Committee of the first affiliated hospital of Harbin medical university and the informed consent was waived.

### 2.2 Data collection

Baseline characteristics, including age, sex, and medical history, were recorded for each patient. Baseline stroke severity was assessed using the NIHSS score. The extent of early cerebral ischemia on baseline CT was measured with the ASPECTS score. Time intervals from stroke onset to groin puncture and to reperfusion were also documented. The location of arterial occlusion was identified using CTA or DSA [internal carotid artery (ICA) or M1 and M2 segments of the middle cerebral artery (MCA)]. Recanalization was measured by the expanded Thrombolysis in Cerebral Infarction (eTICI) score [ranging from 0 (no reperfusion) to 3 (complete reperfusion)] on the final DSA after the procedure, with successful recanalization defined as eTICI 2b-3. Postprocedural symptomatic intracranial hemorrhage (SICH) and asymptomatic intracranial hemorrhage (aSICH) were recorded, and hemorrhage was classified according to the Heidelberg criteria (14). Functional outcomes at 90 days post-stroke were evaluated by neurointerventionists during routine follow-up visits using the modified Rankin Scale (mRS) score. If a patient was unable to return to the hospital, outcomes were assessed through a standardized telephone interview.

### 2.3 Treatment

According to international guidelines, patients in the bridging group received alteplase at a total dose of 0.9 mg/kg within 4.5 h of symptom onset (10% administered as an intravenous bolus and the remaining 90% as an intravenous infusion over 1 h). The infusion could be completed during endovascular treatment. For endovascular mechanical thrombectomy, patients were positioned supine, and either local or general anesthesia was administered based on the patient's cooperation and vascular condition. Cerebral angiography was first performed to assess the site of vascular occlusion and primary compensatory flow. The loach guide wire guided the 8F guide catheter (Boston Scientific, USA) and the intermediate catheter to the vicinity of the affected common carotid artery bifurcation. The intermediate catheter was initially advanced through the severe stenosis or occlusion in the ICA's proximal segment. If unsuccessful, a microcatheter and micro guide wire were used to navigate the ICA under roadmap guidance. Following percutaneous transluminal angioplasty of the ICA, the intermediate catheter was passed through the stenotic or occluded segment, and intracranial vascular thrombectomy and recanalization were performed using standard techniques. After intracranial vessel recanalization, patients were monitored for 20 min, with the degree of stenosis assessed via common carotid artery angiography to determine the need for carotid stent implantation.

### 2.4 Outcomes

The primary outcome was prespecified as a favorable outcome (mRS score of 0–2) at 90 days. Secondary outcomes included successful reperfusion (eTICI score of 2b-3) at the procedure's end, incidence of SICH and aSICH, and overall mortality at 90 days.

### 2.5 Statistical analyses

Continuous variables following a normal distribution were described as mean ± standard deviation (SD), while non-normally distributed data were represented by median and interquartile range (IQR). Categorical variables were presented as numbers with percentages. For continuous variables, independent sample *t*-tests or Mann–Whitney *U*-tests were used according to data distribution, and Fisher's exact test and chi-square test were used for categorical variables. The association between treatment methods and outcomes was expressed as an odds ratio (OR) with adjustments for confounders, estimated by a multivariate logistic regression model. Outcomes were calculated with a 95% confidence interval (CI), and variables with *P* < 0.1 in the univariate analysis were included in the model. Given the number of events, variables were selected cautiously for model entry. All regression results presented both unadjusted and adjusted OR (aOR) values. Statistical analyses were performed using SPSS 22.0 software (IBM), with *P* < 0.05 considered statistically significant.

## 3 Results

### 3.1 Study population

A total of 110 patients were included in the study, with 49 patients assigned to direct mechanical thrombectomy (MT-alone group) and 61 patients to intravenous alteplase followed by MT (bridging group). Key baseline characteristics were as follows: the mean age was 65.6 ± 7.3 years in the MT-alone group and 66.0 ± 7.4 years in the bridging group; baseline median NIHSS scores were 16 (IQR 14–18) and 15 (IQR 12–18), respectively; baseline median ASPECT scores were 8 (IQR 7–9) for both groups. Table 1 presents detailed baseline characteristics for both groups, with no statistically significant differences between them.

**Table 1:** Baseline characteristics of patients with anterior circulation tandem occlusions.

Characteristics	MT-alone group *n* = 49	Bridging group *n* = 61	*P*-value
Sex (male), *n* (%)	38 (77.5)	54 (88.5)	0.122
Age, year, median ± SD	65.6 ± 7.3	66.0 ± 7.4	0.769
Atrial fibrillation, *n* (%)	6 (12.2)	6 (9.8)	0.687
Diabetes mellitus, *n* (%)	14 (28.6)	15 (24.6)	0.638
Hypertension, *n* (%)	23 (46.9)	36 (59.0)	0.207
Hypercholesterolemia, *n* (%)	15 (30.6)	16 (26.2)	0.612
Previous ischemic stroke, *n* (%)	9 (18.4)	15 (24.6)	0.432
Current smoking, *n* (%)	29 (59.2)	32 (52.5)	0.481
Current alcohol abused, *n* (%)	14 (28.6)	20 (32.8)	0.634
mRS before stroke onset, *n* (%)			0.238
0	45 (91.8)	60 (98.4)	
1	3 (6.1)	1 (1.6)	
2	1 (2.0)	0	
Corresponding occlusion cerebral hemisphere, *n* (%)			0.175
Right	17 (34.7)	29 (47.5)	
Left	32 (65.3)	32 (52.5)	
Occlusion site, *n* (%)			0.656
ICA	16 (32.7)	23 (37.7)	
M1	29 (59.2)	31 (50.8)	
M2	4 (8.2)	7 (11.5)	
Stent thrombectomy, *n* (%)	47 (95.9)	59 (96.7)	0.823
Times of thrombectomy, median (IQR)	1 (1, 2)	1 (1, 2)	0.443
Balloon Dilatation, *n* (%)	32 (65.3)	44 (72.1)	0.441
Intraprocedural ICA stent implantation, *n* (%)	27 (55.1)	43 (70.5)	0.095
Decompressive craniectomy, *n* (%)	1 (2.0)	2 (3.3)	1.000
eTICI score, *n* (%)			0.613
0	48 (98.0)	58 (95.1)	
1	1 (2.0)	2 (3.3)	
2a	0	1 (1.6)	
Intraprocedural tirofiban administration, *n* (%)	40 (81.6)	45 (73.8)	0.328
Postprocedural tirofiban administration, *n* (%)	36 (73.5)	43 (70.5)	0.730
Intraprocedural antihypertensive drugs administration, *n* (%)	17 (34.7)	32 52.5)	0.062
Postprocedural dual antiplatelet therapy, *n* (%)	35 (71.4)	49 (80.3)	0.275
Baseline NIHSS, median (IQR)	16 (14, 18)	15 (12, 18)	0.224
ASPECTS, median (IQR)	8 (7, 9)	8 (7, 9)	0.083
From admission to groin puncture, min, median (IQR)	107 (79, 143)	129 (90, 177)	0.087
From stroke onset to reperfusion or last, min, median (IQR)	245 (195, 350)	285 (240, 330)	0.211
From stroke onset to groin puncture, min, median (IQR)	170 (135, 263)	208 (165, 264)	0.140
Treatment strategy, *n* (%)			0.491
Antegrade approach	20 (40.8)	21 (34.4)	
Retrograde approach	29 (59.2)	40 (65.6)	

MT, mechanical thrombectomy; IQR, interquartile range; eTICI, expanded thrombolysis in cerebral infarction; NIHSS, National Institutes of Health Stroke Scale; ASPECTS, Alberta Stroke Program Early CT Score; ICA, internal carotid artery.

### 3.2 Procedure-related data

After perfusion, cervical ICA stenting was performed in 27 (55.1%) patients in the MT-alone group and 43 (70.5%) in the bridging group. The median time from admission to groin puncture was 107 min (IQR 79–143) in the MT-alone group and 129 min (IQR 90–177) in the bridging group. The median time from stroke onset to reperfusion was 245 min (IQR 195–350) for the MT-alone group and 285 min (IQR 240–330) for the bridging group. The median time from stroke onset to groin puncture was 170 min (IQR 135–263) for the MT-alone group and 208 min (IQR 165–264) for the bridging group. Tirofiban was administered intraprocedurally in 40 (81.6%) patients in the MT-alone group and in 45 (73.8%) patients in the bridging group. Post-procedure, tirofiban was administered to 36 (73.5%) patients in the MT-alone group and 43 (70.5%) patients in the bridging group. A total of 28 (57.1%) patients in the MT-alone group and 43 (70.5%) patients in the bridging group received intraprocedural ICA stent implantation. Postprocedural dual antiplatelet therapy was administered to 35 (71.4%) patients in the MT-alone group and 49 (80.3%) patients in the bridging group. Balloon dilatation was performed in 76 patients: 32 (65.3%) in the MT-alone group and 44 (72.1%) in the bridging group. In terms of thrombectomy methods, 47 (95.9%) patients in the MT-alone group and 59 (96.7%) in the bridging group received stent thrombectomy.

### 3.3 Primary outcomes

**Figure 2:**
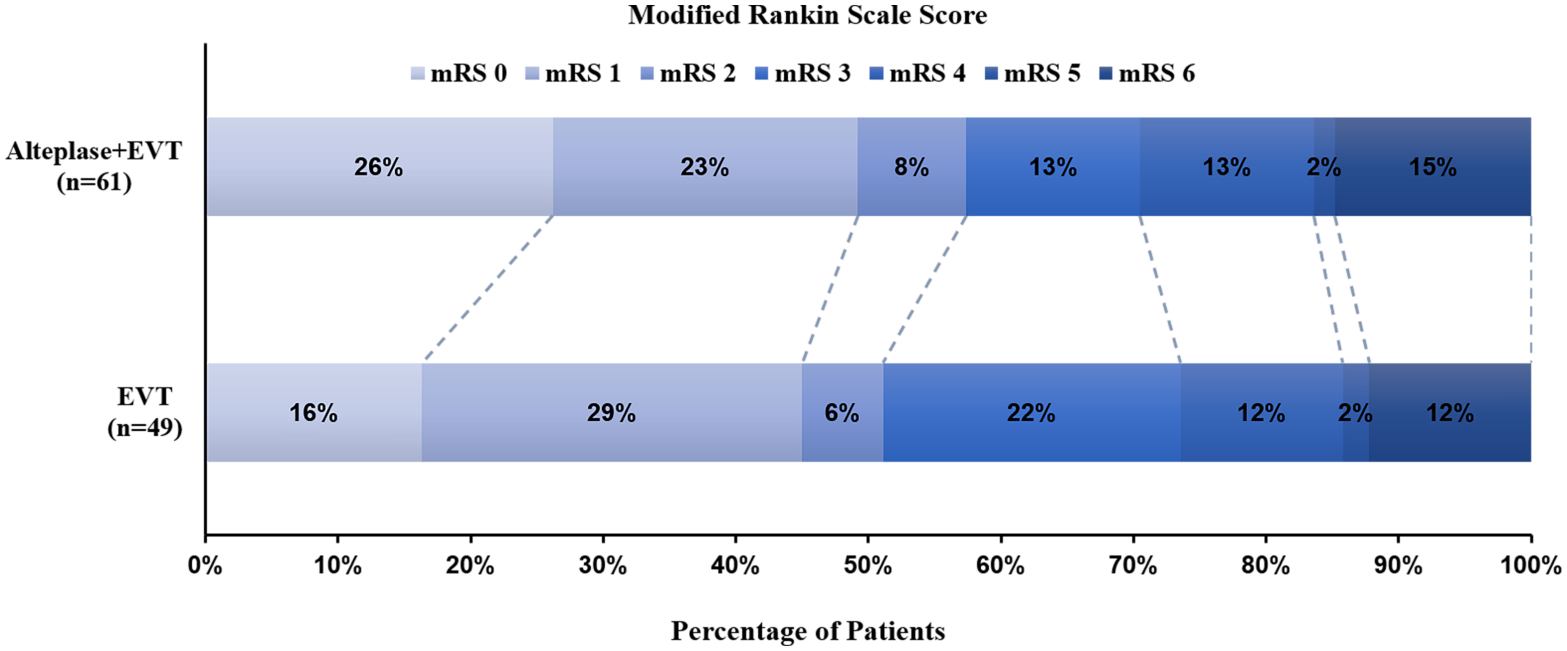
Distribution of modified rankin scale (mRS) scores at 90 days post-treatment. Scores range from 0 to 6, with (0) indicating no symptoms; (1) no significant disability; (2) mild disability (patient can manage self-care independently but cannot perform all previous activities); (3) moderate disability (patient requires some assistance but can walk unassisted); (4) moderately severe disability (patient cannot attend to physical needs or walk without assistance); (5) severe disability (patient requires constant care and attention); and (6) death. The mRS scores at 90 days were comparable between the MT-alone group and the bridging group (*P* = 0.579).

The mRS score at 90 days was comparable between the bridging and MT-alone groups (*P* = 0.579) (Figure 2). A favorable outcome (mRS of 0–2) at 90 days was observed in 25 (51.0%) patients in the MT-alone group and 34 (55.7%) patients in the bridging group, showing no significant difference between the groups (*P* = 0.622) with an unadjusted OR of 1.21 (95% CI, 0.57–2.57). Multivariate analysis found no association between prior intravenous alteplase administration and 90-day functional outcomes in patients with anterior circulation tandem occlusions after adjusting for confounding variables, yielding an aOR of 1.17 (95% CI, 0.47–2.90; adjusted *P* = 0.743) (Table 2).

**Table 2:** Treatment outcomes of patients with anterior circulation tandem occlusions.

Outcome	MT-alone group *n* = 49	Bridging group *n* = 61	Unadjusted		Adjusted	
			Odds ratio (95% CI)	*P*-value	Odds ratio (95% CI)	*P*-value
Primary outcome						
mRS of 0–2 at 90 d, *n* (%)^a^	25 (51.0)	34 (55.7)	1.21 (0.57–2.57)	0.622	1.17 (0.47–2.90)	0.743
Secondary outcomes						
SICH, *n* (%)^b^	4 (8.2)	6 (9.8)	1.23 (0.33–4.62)	0.762	1.80 (0.37–8.62)	0.460
aSICH, *n* (%)^c^	7 (14.3)	19 (31.1)	2.71 (1.03–7.13)	0.039	2.86 (1.04–7.88)	0.042
Death, *n* (%)^d^	6 (12.2)	9 (14.8)	1.24 (0.41–3.76)	0.703	1.22 (0.27–5.46)	0.792
eTICI ≥2b after Reperfusion, *n* (%)^e^	36 (73.5)	51 (83.6)	1.84 (0.73–4.66)	0.194	1.79 (0.66–4.87)	0.257

MT, mechanical thrombectomy; mRS, modified Rankin Scale; SICH, symptomatic intracerebral hemorrhage; aSICH, asymptomatic intracranial hemorrhage; eTICI, Expanded thrombolysis in Cerebral Infarction.

Values were adjusted for the following:.

^a^ Intraprocedural tirofiban administration, intraprocedural carotid artery stent implantation, baseline stroke severity (National Institutes of Health Stroke Scale score), postprocedural tirofiban administration, number of thrombectomies, and balloon dilatation.

^b^ Baseline stroke severity, time from stroke onset to reperfusion, and number of thrombectomies.

^c^ Age, number of thrombectomies, and occlusion site.

^d^ Atrial fibrillation, smoking, occlusion site, decompressive craniectomy, baseline stroke severity, procedure duration, and number of thrombectomies.

^e^ Smoking, affected cerebral hemisphere, and intraprocedural carotid artery stent implantation.

**Figure 3:**
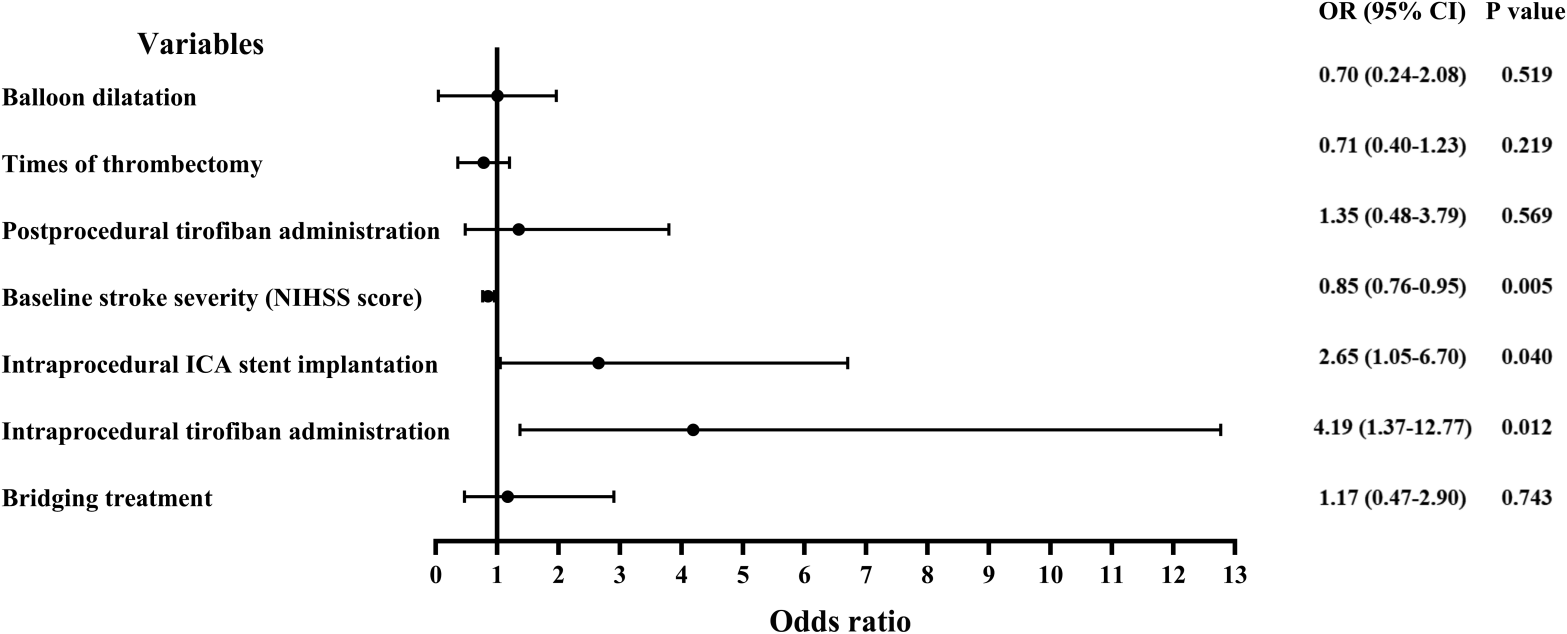
Forest plots demonstrating risk factors for a favorable outcome (mRS score of 0–2) at 90 days post-treatment. OR, odds ratio; ICA, internal carotid artery; NIHSS, National Institutes of Health Stroke Scale.

A subgroup analysis of the primary outcome was conducted using multivariate logistic regression, evaluating intra- or postprocedural tirofiban administration, intravenous alteplase administration before MT, intraprocedural stent implantation, baseline NIHSS score, time from stroke onset to reperfusion, number of thrombectomies, balloon dilation, and procedure duration. Results indicated that intraprocedural tirofiban administration (OR = 4.19, 95% CI, 1.37–12.77; *P* = 0.012), intraprocedural stent implantation (OR = 2.65, 95% CI, 1.05–6.70; *P* = 0.040), and baseline NIHSS score (OR = 0.85, 95% CI, 0.76–0.95; *P* = 0.005) were associated with a favorable outcome at 90 days (Figure 3).

### 3.4 Secondary outcomes

The rate of aSICH was higher in the bridging group than in the MT-alone group, with an aSICH rate of 14.3% in the MT-alone group vs. 31.1% in the bridging group, resulting in an aOR of 2.86 (95% CI, 1.04–7.88; adjusted *P* = 0.042). The rate of SICH was similar between groups (8.9% vs. 9.8%; aOR = 1.80, 95% CI, 0.37–8.62; adjusted *P* = 0.460). The 90-day mortality rate was 12.2% in the MT-alone group and 14.8% in the bridging group, with no significant difference (aOR = 1.22, 95% CI, 0.27–5.46; adjusted *P* = 0.792). The successful recanalization rate was numerically higher in the bridging group (83.6% vs. 73.5%; aOR = 1.79, 95% CI, 0.66–4.87; adjusted *P* = 0.257), though this difference was not statistically significant.

## 4 Discussion

Our study found no evidence that prior intravenous alteplase administration before MT influenced the 90-day outcomes of patients with acute ischemic stroke due to anterior circulation tandem occlusions. However, we observed that bridging therapy was associated with an increased rate of aSICH following the procedure. Although this study did not demonstrate a significant improvement in functional outcomes, MT alone appeared to yield nominally better efficacy outcomes compared to combination therapy.

Previous studies comparing the safety and efficacy of bridging therapy vs. MT alone have reported conflicting results, creating uncertainty regarding the benefits of intravenous thrombolysis before MT (6–10). Recent RCTs, however, have provided robust evidence for the non-inferiority of MT alone compared to combined intravenous alteplase and MT (11, 13). The DIRECT-MT (Endovascular Thrombectomy with or without Intravenous Alteplase in Acute Stroke) trial (11) and the DEVT (Direct Endovascular Thrombectomy vs. Combined IVT and Endovascular Thrombectomy for Patients with Acute Large Vessel Occlusion in the Anterior Circulation) trial (13) demonstrated that MT alone was not inferior to bridging therapy in terms of 90-day neurological function outcomes. Conversely, the SKIP (Effect of Mechanical Thrombectomy Without vs. With Intravenous Thrombolysis on Functional Outcome Among Patients with Acute Ischemic Stroke) trial (12) failed to demonstrate non-inferiority regarding favorable functional outcomes in patients treated with endovascular thrombectomy with or without intravenous alteplase in acute large vessel occlusion stroke. The inconsistency among these RCTs remains unexplained, and all three trials lack specific outcome data for patients with tandem occlusion.

Tandem occlusion is a known risk factor for poor outcomes following intravenous thrombolysis (1, 5). It remains uncertain whether patients with tandem occlusion should undergo intravenous thrombolysis before endovascular treatment. Previous animal studies have shown that downstream microvascular thrombosis is common in large artery occlusion and that early administration of alteplase may improve outcomes post-recanalization by reducing fibrinogen-dependent platelet aggregation (15). Pikija et al. (16) conducted a retrospective study on patients who underwent endovascular therapy for tandem occlusion in acute ischemic stroke, finding that intravenous t-PA administration was associated with a favorable outcome at three months (OR 12.0, 95% CI 1.0–144.4). Similarly, results from Anadani et al. (17) indicated that patients who received intravenous thrombolysis before MT had higher rates of favorable outcomes and successful reperfusion. However, our study yielded conflicting results, with comparable 90-day mRS scores between the bridging and MT-alone groups. This discrepancy may stem from differences in patient selection criteria; studies that included MT alone for patients outside the time window or contraindicated for alteplase may have experienced higher unfavorable outcome rates.

In our study, the overall ICH rate was comparable to that in published trials (11–13). However, regarding secondary safety outcomes, we found that aSICH rates were significantly higher in patients who received intravenous alteplase, with 14.3% in the MT-alone group vs. 31.1% in the bridging group, while SICH rates did not significantly differ between the two groups. The SKIP trial similarly found a lower rate of any ICH in the MT-alone group compared to the combined group (12). Alteplase administration is known to increase the risk of ICH compared to placebo or open control in patients with acute ischemic stroke, potentially leading to more than a five-fold increase in hemorrhage frequency (18). This may explain the higher frequency of aSICH observed in our study. Van Kranendonk et al. (19) demonstrated that hemorrhagic transformation is associated with functional outcomes following acute ischemic stroke. Thus, both SICH and aSICH remain critical and warrant vigilance. Moreover, while the bridging group demonstrated a numerically higher rate of successful reperfusion (eTICI ≥2b) compared to the MT-alone group, this difference did not reach statistical significance (*P* = 0.257). Preclinical studies have established that downstream microvascular thrombosis represents a pathognomonic feature of large artery occlusion, with early alteplase administration demonstrating improved post-recanalization outcomes via fibrinogen-mediated attenuation of platelet aggregation (15). The technical complexity of endovascular thrombectomy in tandem lesions carry risks of post-recanalization re-occlusion, where pre-treatment with rt-PA thrombolysis might provide protective benefits against re-occlusion. Nevertheless, our findings provide no conclusive evidence that bridging treatment enhances post-thrombectomy favorable recanalization rates, warranting validation through larger multicenter cohorts.

We observed that intraprocedural tirofiban administration was associated with favorable 90-day outcomes. Tirofiban has been shown to be safe and effective in endovascular treatments for cerebrovascular diseases, including ruptured and unruptured aneurysms, symptomatic intracranial atherosclerotic stenosis, and acute ischemic stroke (20–23). As a glycoprotein IIb/IIIa inhibitor, tirofiban acts quickly post-administration, and platelet function can be promptly reversed after discontinuing the infusion. Tirofiban administration may prevent thrombus formation and reduce ischemic events (22). Additionally, our study indicated that intraprocedural stent implantation was linked to a higher favorable outcome rate at 90 days. Acute stent implantation for extracranial carotid stenosis is a crucial management approach for tandem lesions in acute ischemic stroke and may enhance clinical outcomes (24). The benefit of acute stent implantation may relate to improved intracranial vascular perfusion post-implantation (22, 24). Acute stent implantation for extracranial carotid stenosis can also stabilize the unstable plaques in the proximal carotid artery, preventing their detachment and subsequent thromboembolic events in distal vessels. Additionally, this approach provides a more favorable vascular pathway for endovascular treatment of distal occlusions, allowing the micro-guidewire and microcatheter to reach the distal occluded vessel under direct visualization, thereby mitigating ischemic events caused by vascular dissection and endothelial injury to some extent.

This study has several limitations. Our sample size was relatively small, resulting in wide confidence intervals when analyzing patient outcomes and reducing the statistical power. Additionally, our study was retrospective and non-randomized, with potential biases (e.g., selection bias) that are unavoidable in retrospective studies. In our study, we did not compare the possible impacts of other intravenous thrombolytic drugs on the outcomes, such as tenecteplase. The absence of comparative analysis between intravenous thrombolysis alone and endovascular therapy regarding safety and efficacy in anterior circulation tandem occlusion also represents a limitation of our study. Moreover, although we used a multivariate analysis model to adjust for primary and secondary outcomes to minimize confounding factor influence, residual confounding factors may still affect our findings. Therefore, a prospective, randomized controlled trial would be valuable.

## 5 Conclusion

Our findings indicated that prior intravenous alteplase administration did not impact the clinical or imaging outcomes of patients with acute ischemic stroke due to anterior circulation tandem occlusions, as reflected by similar mRS scores at 90 days. Bridging therapy, however, was associated with a higher rate of aSICH. Favorable outcomes were associated with intraprocedural tirofiban administration, stent implantation, and a lower baseline NIHSS score.

## Data Availability

The raw data supporting the conclusions of this article will be made available by the authors, without undue reservation.
